# Usefulness of open lung biopsy in mechanically ventilated patients with undiagnosed diffuse pulmonary infiltrates: influence of comorbidities and organ dysfunction

**DOI:** 10.1186/cc6106

**Published:** 2007-08-28

**Authors:** Seong Yong Lim, Gee Young Suh, Jae Chol Choi, Won Jung Koh, Si Young Lim, Joungho Han, Kyung Soo Lee, Young Mog Shim, Man Pyo Chung, Hojoong Kim, O Jung Kwon

**Affiliations:** 1 Division of Pulmonary and Critical Care Medicine, Department of Medicine, Kangbuk Samsung Hospital, Sungkyunkwan University School of Medicine, 108 Pyeong-dong, Jongno-gu, Seoul, South Korea, 110-746; 2 Division of Pulmonary and Critical Care Medicine, Department of Medicine, Samsung Medical Center, Sungkyunkwan University School of Medicine, 50 Irwon-dong, Gangnam-gu, Seoul, South Korea, 135-710; 3 Department of Pathology, Samsung Medical Center, Sungkyunkwan University School of Medicine, 50 Irwon-dong, Gangnam-gu, Seoul, South Korea, 135-710; 4 Department of Radiology, Samsung Medical Center, Sungkyunkwan University School of Medicine, 50 Irwon-dong, Gangnam-gu, Seoul, South Korea, 135-710; 5 Department of Thoracic Surgery, Samsung Medical Center, Sungkyunkwan University School of Medicine, 50 Irwon-dong, Gangnam-gu, Seoul, South Korea, 135-710

## Abstract

**Background:**

The purpose of this study was to evaluate the clinical usefulness of open lung biopsy (OLB) in patients undergoing mechanical ventilation for diffuse pulmonary infiltrates of unknown etiology.

**Methods:**

This was a 10-year retrospective study in a 10-bed medical intensive care unit. The medical records of 36 ventilator-dependent patients who underwent OLB for the diagnosis of unknown pulmonary infiltrates from 1994 to 2004 were reviewed retrospectively. Data analyzed included demographic data, Charlson age–comorbidity score, number of organ dysfunctions, Acute Physiology and Chronic Health Evaluation (APACHE) II, Simplified Acute Physiology Score (SAPS) II, Sequential Organ Failure Assessment (SOFA) score, ventilation variables, and radiological patterns. Diagnostic yield, effect on subsequent treatment changes, and complications of OLB were also assessed.

**Results:**

A specific clinico-pathologic diagnosis was obtained for 31 patients (86%). The most common diagnoses were interstitial pneumonia (*n *= 17, including 8 acute interstitial pneumonia) and viral pneumonia (*n *= 4). Therapeutic modifications were made in 64% of patients. Patients who received OLB less than 1 week after initiation of mechanical ventilation were more likely to survive (63% versus 11%; *P *= 0.018). There were no major complications associated with the procedure. Factors independently associated with survival were the Charlson age-comorbidity score, number of organ dysfunction and the PaO_2_/FiO_2 _ratio on the day of the OLB.

**Conclusion:**

OLB can provide a specific diagnosis in many ventilator-dependent patients with undiagnosed pulmonary infiltrate. Early OLB seems to be useful in critically ill patients with isolated respiratory failure.

## Introduction

The development of progressive pulmonary infiltrates in a patient with respiratory failure is a challenging situation for the clinician. Although open lung biopsy (OLB) remains the gold standard for the diagnosis of parenchymal lung disease [1-3], it is unclear whether the results obtained from an OLB are truly beneficial to these critically ill patients. Whereas some authors [2,4,5] have noted that OLB is safe as well as diagnostically useful, permitting the institution of appropriate therapy, some [6,7] argue against the usefulness of OLB because it may be associated with substantial morbidity and mortality. Because of these potential harmful effects, many clinicians have been reluctant to perform OLB in patients who are already ventilator-dependent. A recent study by Papazian and colleagues [8] demonstrated that OLB improved the survival of patients with unresolving acute respiratory distress syndrome (ARDS) when biopsy findings were contributory. However, whether OLB is helpful in patients with diffuse lung infiltrates of unknown etiology who are sick enough to warrant ventilator therapy is still controversial. This study was therefore undertaken to assess the usefulness and safety of OLB and to identify the prognostic factors associated with survival in ventilator-dependent patients with diffuse pulmonary infiltrates of unknown origin.

## Materials and methods

We conducted a retrospective review of the clinical records of patients admitted to an adult medical intensive care unit (ICU) from October 1994 to October 2004 at Samsung Medical Center. The inclusion criteria were patients with respiratory failure who underwent OLB as a result of undiagnosed diffuse pulmonary infiltrates while receiving mechanical ventilatory support. Patients who did not need mechanical ventilation or patients who started ventilatory support after OLB were excluded. Over the period examined, 513 surgical lung biopsies were performed for diagnostic purposes at our institution. For surgical lung biopsy, elective video-assisted thoracoscopic surgery was used in 381 patients (74%), and OLB via minithoracotomy was conducted in 133 (26%). In all, 42 patients underwent surgical lung biopsy for respiratory failure of unknown etiology. Six patients were excluded because they were not on mechanical ventilatory support at the time of the procedure, and 36 patients met our inclusion criteria. None of the patients included in this study underwent video-assisted thoracoscopic surgery as the method of lung biopsy.

The medical records from the 36 cases above were analyzed for the following data: demographic data, body mass index, comorbidities, time from mechanical ventilation to OLB, radiological findings, ventilation variables including the PaO_2_/FiO_2 _ratio, the positive end-expiratory pressure (PEEP), and compliance; in addition, severity scores such as Simplified Acute Physiology Score (SAPS) II, Acute Physiology and Chronic Health Evaluation (APACHE) II and Sequential Organ Failure Assessment (SOFA) score were analyzed. The SOFA score on the day of ICU admission (SOFA_adm_), on the day of OLB (SOFA_olb_), and the maximum score before the OLB (SOFA_max_) were assessed. The number of organ dysfunctions represented the number of organs that scored more than 2 points on the SOFA score. We also collected data on previous diagnostic studies and their results, preoperative therapeutic measures, pathology, perioperative complications, the effect of OLB on patient management, and the resultant outcome at ICU discharge. Life-threatening major complication was defined as the occurrence of death, myocardial infarction, or stroke within 48 hours of surgery. Documented hypoxia (arterial oxygen saturation less than 90%), hypotension or arrhythmia requiring intervention during the procedure was recorded. Prolonged air leakage for more than 1 week, wound infection, bleeding events or any other complications thought to be directly related to the procedure were also documented. Overall comorbidity was assessed with the Charlson age–comorbidity score (CCS) [9].

Our typical OLB protocol was as follows. All OLBs were conducted in the operating room under general anesthesia by means of anterior minithoracotomy. Sites for pulmonary biopsy were selected before surgery by a thorough review of chest radiographs and computed tomography studies. After multiple wedge biopsies, drainage of the pleural space was performed with a chest tube. Generally, these tubes were removed as soon as possible if no air leak was present. The operative time including anesthesia averaged about 1 hour. The lung biopsy specimens were submitted for bacterial, fungal, acid-fast bacillus and viral cultures as well as histological examination. The final diagnosis was made on the basis of a correlation of the clinical and pathological findings.

Statistical analysis was performed with SPSS v.13.0 package for Windows (SPSS Inc., Chicago, IL, USA). Results are expressed as mean ± SD. Survivors and non-survivors were compared by using the independent-sample *t *test for continuous variables, and the χ^2 ^test or Fisher's exact test for categorical variables. Univariate analysis was performed and a relative risk with a 95% confidence interval (CI) was determined. To assess the factors related to survival, multiple-logistic-regression analysis was performed, with ICU discharge as the dependent variable. For all statistical tests used, *P *< 0.05 was considered significant.

## Results

### Patient characteristics before OLB

Characteristics of patients are shown in Table 1. Of the 36 patients enrolled in the study, 25 were male (69%) and 11 were female (31%), with a mean age of 58.5 years (range 20 to 77). The mean CCS was 2.6 (range 0 to 7). The mean number of organ dysfunctions was 2 (range 1 to 3), SOFA_adm _was 5 (range 2 to 12) and the PaO_2_/FiO_2 _ratio was 119.5 (range 53 to 267). The median time from mechanical ventilatory support to OLB was 4 days (range 1 to 23). Preexisting comorbid diseases were found in 19 patients (53%).

**Table 1:** Assessment of patient characteristics on admission and on the day of open lung biopsy

Characteristic	On admission to ICU	On day of OLB
Age, years	58.5 (20–77)	
Sex		
Male	25 (69)	
Female	11 (31)	
Smoking, pack-years	20 (0–80)	
CCS	2.6 (0–7)	
APACHE II score	17 (7–27)	
SAPS II score	35 (22–65)	
SOFA score	5 (2–12)	5 (1–12)
No. of organ dysfunctions	2 (1–3)	2 (1–3)
PaO_2_/FiO_2 _ratio	119.5 (53–267)	158.6 (52–320)
PEEP, cmH_2_O	10 (5–18)	10 (5–18)
Compliance, ml/cmH_2_O	14.9 (5.1–36.3)	14.0 (5.2–39.3)

Data are presented as mean (range) or *n *(%). CCS, Charlson age–comorbidity score; APACHE II, Acute Physiologic and Chronic Health Evaluation II; ICU, intensive care unit; OLB, open lung biopsy; PEEP, positive end-expiratory pressure; SAPS, Simplified Acute Physiology Score; SOFA, Sequential Organ Failure Assessment.

### Preoperative diagnostic procedures and therapeutic measures

Preoperative fiberoptic bronchoscopic examination and bronchoalveolar lavage (BAL) was performed in 31 patients (86%). BAL revealed positive staining for acid-fast bacilli in two patients who were already receiving anti-tuberculous medication for previously diagnosed tuberculosis and were undergoing diagnostic study for progressive lung infiltrates while on adequate anti-tuberculous medications. Two other patients had progressive hemorrhagic BAL consistent with diffuse alveolar hemorrhage. In the other cases the BAL was not helpful. Of the radiological studies, diffuse ground glass opacity alone or combined with consolidation was the predominant radiological finding in most of the patients (28/36; 78%).

At the time of OLB, all 36 patients were receiving empirical antibiotic treatment. Eleven patients were on antibiotics only but the rest were receiving combination therapy with one or more agents: 18 were receiving steroids, 4 antiviral agents, 3 anti-tuberculous medication and 2 antifungal drugs.

### Results of open lung biopsy and effect on patient management or outcome

A specific clinico-pathologic diagnosis as a cause of progressive pulmonary infiltrates was established in 31 patients (86%) after OLB. The specific clinico-pathologic diagnosis based on the OLB is shown in Table 2. The most common diagnosis obtained was interstitial pneumonia (*n *= 17). Fifteen patients were idiopathic and two had secondary interstitial pneumonia. Idiopathic interstitial pneumonia included acute interstitial pneumonia (AIP) (*n *= 8), cryptogenic organizing pneumonia (*n *= 3), acute exacerbation of usual interstitial pneumonia (*n *= 3) and nonspecific interstitial pneumonia group 3 (*n *= 1). AIP was finally diagnosed after ruling out other factors that could cause diffuse alveolar damage. Secondary interstitial pneumonia included one patient with non-tuberculous mycobacterium-associated bronchiolitis obliterans organizing pneumonia and one with dermatomyositis-associated acute pneumonitis. The most common alternative diagnosis other than interstitial pneumonia was viral pneumonia (*n *= 4), including two cases of cytomegalovirus (CMV) and another two cases of adenovirus pneumonia. Two patients each had drug toxicity due to chemotherapeutic agents, miliary tuberculosis and idiopathic pauci-immune pulmonary capillaritis. Other diagnoses included cholesterol crystal embolism, culture-negative bacterial pneumonia, diffuse panbronchiolitis, and metastatic cancer.

**Table 2:** Specific clinico-pathologic diagnosis obtained from 31 patients

Diagnosis	No. of patients
Idiopathic interstitial pneumonia	15
Acute interstitial pneumonia	8
Cyptogenic organizing pneumonia	3
Acute exacerbation of usual interstitial pneumonia	3
Nonspecific interstitial pneumonia group 3	1
Secondary interstitial pneumonia	2
Non-tuberculous mycobacterium-associated BOOP	1
Dermatomyositis-associated acute pneumonitis	1
Viral pneumonia	4
Cytomegalovirus pneumonia	2
Adenovirus pneumonia	2
Miliary tuberculosis	2
Chemotherapy drug toxicity	2
Idiopathic pauci-immune pulmonary capillaritis	2
Diffuse panbronchiolitis	1
Cholesterol crystal embolism	1
Acute necrotizing pneumonia	1
Metastatic cancer	1

BOOP, bronchiolitis obliterans organizing pneumonia.

Twenty-three patients (64%) were able to change their therapy on the basis of the OLB results. Drug changes usually involved the initiation of steroids (*n *= 15) or antiviral agents (*n *= 3). Withdrawal of unnecessary medication was possible in two patients. The percentage of patients who received therapeutic modifications was not different between survivors and non-survivors.

### Complications

Twenty patients (56%) had complications that may have been related to OLB. Prolonged air leakage was a predominant complication present in 15 patients (42%). We noted another five cases of intraoperative complications (14%), including four cases of transient hypotension and one of transient hypotension with bigeminy requiring lidocaine treatment. However, there were no life-threatening complications associated with the procedure. There was no statistically significant factor that predicted the occurrence of complication of OLB (data not shown).

### Comparison between survivors and non-survivors

The overall mortality rate in the ICU for this patient population was 50%. Table 3 shows a comparison of the clinical characteristics between survivors and non-survivors. There were no significant differences in a variety of measures including age, sex, body mass index, smoking history, respiratory symptom duration, incidence of OLB complications, immune status, SAPS II score, APACHE II score, time to OLB, serum albumin, and serum glucose.

**Table 3:** A comparison between survivors and non-survivors

Characteristic	Survivor group (*n *= 18)	Non-survivor group (*n *= 18)	*P*
Age, years	54.5 ± 14.7	57.3 ± 15.2	0.580
Sex			
Male	11 (61.1)	14 (77.8)	0.471
Female	7 (38.9)	4 (22.2)	
BMI, kg/m^2^	22.9 ± 2.3	21.7 ± 3.1	0.283
Smoking, pack-years	21.8 ± 23.6	19.9 ± 20.7	0.873
Duration of symptom, days	21.7 ± 33.5	15.2 ± 13.1	0.888
CCS	1.9 ± 1.3	3.2 ± 2.1	0.030
SAPS II score	35.2 ± 9.3	37.6 ± 7.9	0.425
APACHE II score	16.9 ± 5.4	17.6 ± 4.9	0.700
Time to OLB, days	3.8 ± 2.0	6.8 ± 6.4	0.061
OLB time, early/late	17/1	10/8	0.018
Immunocompromised status	3 (17)	7 (39)	0.137
OLB complication	8 (44)	12 (67)	0.180
Ventilation duration, days	14.9 ± 15.5	19.9 ± 12.0	0.282
Number of organ dysfunctions			
On MICU admission	1.5 ± 0.6	1.7 ± 0.7	0.308
On day of OLB	1.5 ± 0.6	2.0 ± 0.8	0.039
SOFA score			
On MICU admission	5.3 ± 2.5	6.2 ± 2.3	0.249
On day of OLB	4.3 ± 2.1	6.7 ± 2.7	0.005
Maximum	6.1 ± 2.7	8.1 ± 2.9	0.042
PaO_2_/FiO_2_ratio			
On MICU admission	131.3 ± 37.1	118.9 ± 55.9	0.439
On day of OLB	190.6 ± 67.6	135.4 ± 57.4	0.012
PEEP, cmH_2_O			
On MICU admission	10.2 ± 3.1	11.3 ± 4.2	0.381
On day of OLB	9.4 ± 4.3	11.3 ± 2.4	0.112
Compliance, ml/cmH_2_O			
On MICU admission	16.7 ± 7.7	15.8 ± 5.5	0.703
On day of OLB	17.1 ± 8.4	15.5 ± 7.5	0.550

Data are presented as mean ± SD or *n *(%). BMI, body mass index; CCS, Charlson age–comorbidity score; SAPS, Simplified Acute Physiology Score; APACHE II, Acute Physiologic and Chronic Health Evaluation II; OLB, open lung biopsy; early OLB, OLB within 1 week of mechanical ventilation; MICU, medical intensive care unit; SOFA, Sequential Organ Failure Assessment; PEEP, positive end-expiratory pressure.

However, the CCS in the non-survivors (3.2 ± 2.1; mean ± SD) was significantly higher than in the survivors (1.9 ± 1.3, *P *= 0.030). Severity and ventilation variables that differed significantly between the two groups were SOFA_olb_, SOFA_max_, PaO_2_/FiO_2 _ratio, and the number of organ dysfunctions on the day of the OLB. Although the mean time from mechanical ventilation to OLB was not different between the two groups, more patients (17/18) in the survivor group received OLB during the early phase, within 1 week of mechanical ventilation, than those in the non-survivor group (10/18, *P *= 0.018).

### Prognostic factors associated with outcome

In univariate analysis, a higher CCS, an increased number of organ dysfunctions, a higher SOFA_olb _score, and a lower PaO_2_/FiO_2 _ratio on the day of the OLB was significantly associated with death (Table 4). A multiple logistic regression analysis showed that a higher CCS (OR 1.74; 95% CI 1.002 to 3.01), an increased number of organ dysfunctions (OR 5.24; 95% CI 1.11 to 24.72), and a lower PaO_2_/FiO_2 _ratio on the day of the OLB (OR 0.98; 95% CI 0.957 to 0.996) were associated with mortality (Table 5).

**Table 4:** Univariate analysis of variables associated with mortality

Variable	*P*	Odds ratio	95% CI
CCS	0.041	1.57	1.02–2.43
Number of organ dysfunctions	0.047	2.85	1.02–8.01
SOFA_olb_	0.013	1.55	1.10–2.19
SOFA_max_	0.055	1.30	0.99–1.70
PaO_2_/FiO_2 _ratioon OLB day	0.023	0.985	0.973–0.998
Time to OLB, days	0.086	1.18	0.98–1.43
OLB complication	0.184	2.50	0.65–9.65
Immunocompromised status	0.146	0.31	0.67–1.50

CCS, Charlson age–comorbidity score; SOFA_olb_, SOFA (Sequential Organ Failure Assessment) score at the day of open lung biopsy; SOFA_max_, maximum SOFA score; OLB, open lung biopsy; time to OLB, days from mechanical ventilation to OLB; CI, confidence interval.

**Table 5:** Multiple logistic regression analysis of variables associated with mortality

Variable	*P*	Odds ratio	95% CI
CCS	0.049	1.74	1.002–3.01
Number of organ dysfunctions	0.036	5.24	1.11–24.72
PaO_2_/FiO_2 _ratioon day of OLB	0.018	0.98	0.957–0.996

Only regressions with *P *< 0.05 are shown; all regression models include age, Charlson age–comorbidity score (CCS), number of organ dysfunctions on the day of open lung biopsy (OLB), PaO_2_/FiO_2 _ratioon the day of OLB, days from mechanical ventilation to OLB, OLB-initiated treatment change, and development of mechanical complications after OLB. CI, confidence interval.

## Discussion

The major findings of this study are that OLB is an feasible diagnostic option even in these critically ill patients and that comorbidity, SOFA score, and PaO_2_/FiO_2 _ratio on the day of the OLB were strong predictors of mortality in these patients. Moreover, although not statistically significant on multivariate analysis, the early, rather than late, use of OLB seems to have a survival advantage.

When a patient is intubated and mechanical ventilation is initiated as a result of respiratory failure of unknown etiology, the clinician is faced with a difficult decision. An invasive diagnostic test such as OLB can be considered but it is not clear which patient subset will benefit from this potentially harmful procedure. In the literature some reports [5,7,10] have looked into the utility of OLB in patients with respiratory failure, but these studies included significant number of patients who were not receiving mechanical ventilatory care and were thus less sick at the time of the biopsies. We therefore performed this study to assess the utility and prognostic factors associated with OLB in patients who were already on mechanical ventilators at the time of the surgical procedure.

One large series of OLB in mechanically ventilated critically ill patients was recently published by Papazian and colleagues [8]. However, the patient population in that study was different from that in this study. The patients in that study all had underlying etiologies for ARDS and underwent OLB only when the lung infiltrates did not resolve. This is a clearly different clinical scenario from that of this study, in which the patients underwent OLB because the cause of lung infiltrate and respiratory failure was unclear. This is reflected by the time of OLB after the initiation of mechanical ventilation, which was a median of 11 days in the study by Papazian and colleagues but only 4 days in our patients.

In this study, a specific clinico-pathologic diagnosis was made in 86% of patients who underwent OLB while on mechanical ventilation before biopsy. In addition, therapeutic changes were made in about two-thirds of patients without life-threatening procedure-related complications. The reported specific diagnostic rate of OLB has varied from 46% [10] to 100% [11]. This discrepancy can be partly explained by the definition for specific diagnosis used in the study. In studies with a high diagnostic rate, pathologic findings consistent with interstitial pneumonitis or alveolitis were regarded as specific diagnoses [11], whereas in studies with a low diagnostic rate these findings were regarded as nonspecific [10]. In the patients in the present study, the specific diagnosis was made in 86% of the biopsied patients by carefully correlating clinical findings with microbiologic and pathologic findings using established criteria, including those for interstitial lung diseases [12]. For example, a pathologic finding of diffuse alveolar damage was critical for the final diagnosis of acute interstitial pneumonia in patients with progressive pulmonary infiltrate who did not have positive microbiologic findings and had no history of exposure to other causes of diffuse alveolar damage.

The role of CMV infection in critically ill patients is still unclear. There are several reports of a high incidence of CMV pneumonia in critically ill patients even in those without overt immunodeficiences [8,13-15]. The relatively high incidence of CMV infection may be explained by the fact that noninvasive diagnostic modalites such as shell-vial culture and CMV pp65 antigenemia have low sensitivity. Although the reported incidence of CMV infection in patients in the ICU showed inconsistent results, our result (6%) was much lower than in a recent report by Papazian and colleagues [8], who demonstrated a high incidence of CMV infection in 30/57 (53%) OLB in unresolving patients with ARDS. It might be that CMV reactivation requires time and the timing of lung biopsies, which was early in the present study (median 4 days versus median 11 days for Papazian and colleagues), might have influenced the results. Further prospective studies are needed to define the clinical significance of CMV and to assess the role of preemptive treatment of antiviral agents.

The low incidence of infectious causes in immunocompromised populations is intriguing. In this study, 10 immunocompromised patients were included (5 with hematologic malignancies, 2 with lung cancer, 1 with systemic lupus erythematosus, and 2 with chronic steroid use), and infectious etiologies were found only 2 patients. This result suggests that the simple use of empirical therapy against infectious organisms might not be enough and that invasive diagnostic tests such as OLB should be actively sought even in immunocompromised patients when they do not respond to empirical therapy. OLB may allow the common use of empirical antibiotics to be tailored or even discontinued if they are not indicated.

Previous studies [4,7,8,10,11,16,17] have noted mortality rates between 38 and 80% in patients with respiratory failure who required OLB. Some of the studies cited a requirement for mechanical ventilation as a strong predictor of poor outcome [7,10,17,18]. In our series, the overall mortality rate in the ICU was 50%. Although it is difficult to compare the overall results because of different study designs and study populations, the mortality in our study was at least comparable with that in previous studies especially given the fact that a requirement for mechanical ventilation was a poor prognostic factor in many of the studies.

Twenty patients (56%) experienced complications related to OLB; these consisted mainly of prolonged postoperative air leakage. Minor complications, including transient hypotension or arrhythmia during operation, occurred in five patients. Our 56% complication rate seems to be slightly higher than those of the studies by Canver and Mentzer [11] (40%), Warner and colleagues [7] (21%) and Flabouris and Myburgh [10] (17%). This higher rate of complications is probably due to the fact that all patients in this study were under mechanical ventilatory support with high PEEP, which predisposes patients to prolonged air leakage. Despite the high incidence of prolonged air leakage, no deaths were directly attributed to the complications from OLB and there was no significant difference in survival rate between those with complications and those without.

Comorbid diseases have been shown to be an important prognostic factor in numerous studies in critically ill patients. The CCS, developed by Charlson and colleagues [9] in 1987, is the sum of 19 predetermined comorbidities given a weighted score of 1, 2, 3, or 6 on the basis of the magnitude of the adjusted relative risk associated with each comorbidity in a Cox proportional hazards regression model. The CCS is a simple score to compute and objectively reflects the seriousness of the combined influence of underlying conditions that may contribute to survival [19]. In the present study, the CCS was shown, in both univariate and multivariate analyses, to be an important prognostic factor. In this study, 71% of those without preexisting comorbidity survived, in contrast with only 32% of those who had preexisting comorbidities. Patients without comorbid diseases might have had a better capacity to withstand the inciting insult, making it easier for them to respond to appropriate therapy. Interestingly, more than half of the survivors without preexisting comorbid diseases were diagnosed with idiopathic interstitial pneumonitis (seven patients had acute interstitial pneumonia, two had cryptogenic organizing pneumonia, and one had fibrotic nonspecific interstitial pneumonia) and responded favorably to high-dose steroid therapy. AIP is known to be a deadly disease with mortality rate of more than 50% [20]. However, recent reports from our group and others show that an early aggressive diagnostic approach, mechanical ventilation with a lung-protective strategy, and the early institution of high-dose steroid pulse therapy may improve the clinical outcome [21,22].

The timing of OLB is controversial. In the present study, although the duration of mechanical ventilation before the OLB did not differ between the two groups, patients who received OLB less than 1 week after the initiation of mechanical ventilation were more likely to survive (63% versus 11%; *P *= 0.018). In the literature there are several reports that also suggest the benefit of early OLB. Warner and colleagues [7] reported that the time from the onset of respiratory failure to OLB was significantly less in survivors (4.4 ± 2.9 days) than in non-survivors (6.1 ± 3.6 days). McKenna and colleagues [23] found that early OLB (average 3.6 days) benefited immunocompromised patients with a histological diagnosis of interstitial pneumonia who were treated with steroids. In addition, Lachapelle and Morin [16] observed that the institution of new therapy was more beneficial in patients who underwent early OLB compared with those undergoing late OLB. Coupled with the fact that, in the present study, the PaO_2_/FiO_2 _ratio and the SOFA score before OLB were significantly worse in the non-survivor group, it seems to be important to perform a biopsy early in the course of disease before irreversible lung parenchymal damage or end-organ damage has set in. This will give the patients the best chance to respond to appropriate therapy. However, because urgent OLB without previous diagnostic tests or empiric therapy does not provide any survival benefit over elective OLB [24], a prudent approach, including initial stabilization and a trial of empirical treatment, seems rational. Further studies on the optimal timing of OLB are needed.

There are several limitations to this study. First, the selection bias may have affected the result of our study. It is possible that patients with more severe disease were excluded because their condition precluded them from biopsy, or patients may have died before biopsy was performed. Second, its retrospective design may have affected the data for several factors. For example, the impact of OLB on therapeutic modification may have been underestimated or even overestimated. Third, the limited sample size in a heterogeneous patient population and the single-institution design of this paper limit the generalization of our findings. Although a prospective randomized study is able to draw powerful conclusions about the role of OLB, it may be very difficult to perform prospective trials in these critically ill patients. A more realistic and potentially useful study design might be a well-constructed matched case-control study, preferably multicentered. These studies will allow us to make better decisions and possibly confirm the benefit of early OLB.

## Conclusion

OLB remains a clinically valuable tool in patients with respiratory failure of unknown etiology, even if it is severe enough to require mechanical ventilatory support. OLB can provide a specific diagnosis in many ventilator-dependent patients with undiagnosed pulmonary infiltrate without life-threatening complications. The early use of OLB seems to be useful in critically ill patients with isolated respiratory failure.

## Key messages

• Factors independently associated with survival were the Charlson age–comorbidity score, the number of organ dysfunctions and the PaO_2_/FiO_2 _ratio on the day of the OLB.

• The survival rate for the patients who underwent OLB at an early stage was better than those who did so at a late stage.

• Prolonged air leakage was the main complication related to OLB. However, no deaths were directly attributable to complications from OLB.

• OLB can provide a specific diagnosis in many ventilator-dependent patients, and early OLB seems to be useful in critically ill patients with isolated respiratory failure.

## Abbreviations

AIP = acute interstitial pneumonia; APACHE II = Acute Physiologic and Chronic Health Evaluation II; ARDS = acute respiratory distress syndrome; BAL = bronchoalveolar lavage; CCS = Charlson age–comorbidity score; CI = confidence interval; CMV = cytomegalovirus; ICU = intensive care unit; OLB = open lung biopsy; PEEP = positive end-expiratory pressure; SAPS = Simplified Acute Physiology Score; SOFA = Sequential Organ Failure Assessment; SOFA_adm _= SOFA score on the day of ICU admission; SOFA_max _= maximum score before the OLB; SOFA_olb _= SOFA score on the day of OLB.

## Competing interests

The authors declare that they have no competing interests.

## Authors' contributions

Seong Yong Lim wrote the protocol, collected data, carried out analyses, and wrote the manuscript. GYS conceived and coordinated the study, and wrote the manuscript. JCC and Si Young Lim collected and analyzed data. JH reviewed the pathologic specimens. KSL helped to review the radiological findings. YMS participated in the design of the study. WJK, MPC, HK, and OJK participated in the design of the study and helped to draft the manuscript. All authors read and approved the final manuscript.
